# Intelligent environments for all: a path towards technology-enhanced human well-being

**DOI:** 10.1007/s10209-021-00797-0

**Published:** 2021-03-15

**Authors:** Laura Burzagli, Pier Luigi Emiliani, Margherita Antona, Constantine Stephanidis

**Affiliations:** 1 “Nello Carrara” Institute of Applied Physics (IFAC), National Research Council (CNR), Sesto Fiorentino, Italy; 2 Institute of Computer Science (ICS), Foundation for Research and Technology–Hellas (FORTH), Heraklion, Crete Greece; 3 Computer Science Department, University of Crete, Heraklion, Crete Greece

**Keywords:** Assistive Intelligent Environments, Design for All, Well-being, Artificial Intelligence, Ethics

## Abstract

Emerging intelligent environments are considered to offer significant opportunities to positively impact human life, both at an individual and at a societal level, and in particular to provide useful means to support people in their daily life activities and thus improve well-being for everybody, especially for older people and for people with limitations of activities. In this context, accessibility and usability, although necessary, are not sufficient to ensure that applications and services are appropriately designed to satisfy human needs and overcome potential functional limitations in the execution of everyday activities fundamental for well-being. This position paper puts forward the claim that, in order to achieve the above objective, it is necessary that: (i) the design of *Assistive Intelligent Environments* is centered around the well-being of people, roughly intended as the possibility of executing the (everyday) human activities necessary for living (independently), thus emphasizing usefulness in addition to usability; (ii) the technological environment is orchestrated around such activities and contains knowledge about how they are performed and how people need to be supported to perform them; (iii) the environment makes use of monitoring and reasoning capabilities in order to adapt, fine-tune and evolve over time the type and level of support provided, and this process takes place considering ethical values; (iv) the applications must also support the possibility of contact with other people, who in many cases may be the only effective help. Moving forward from the *Design for All* paradigm, this paper discusses how the latter can be revisited under the perspective of technology’s usefulness and contribution to human well-being. Subsequently, it introduces a practical notion of well-being based on the ICF classification of human functions and activities and discusses how such notion can constitute the starting point and the focus of design approaches targeted to assist people in their everyday life mainly (but not exclusively) in the home environment. As a subsequent step, the need for integrating Artificial Intelligence capabilities in assistive intelligent environments is discussed, based on the complexity of the human problems to be addressed and the diversity of the types of support needed. The proposed approach is exemplified and illustrated through the experience acquired in the development of four applications, addressing vital aspects of human life, namely *nutrition*, *stress management*, *sleep management* and *counteracting loneliness*. Finally, based on the acquired experience, the need to take into account ethical values in the development of assistive intelligent environments is discussed.

## Introduction

The current evolution of ICT is leading towards the development of heterogeneous technologies, including smart (computer-based) devices and objects, that are highly interconnected and cooperate over the Internet, creating technology-enriched environments where interaction occurs at various levels between humans, devices, and autonomous agents. Communication across the Internet also allows the cooperation of devices and physical objects across different living environments, as foreseen in the development of the concept of the Internet of Things [3]. Additionally, the rapid evolution of Artificial Intelligence also promises to make new technologies intelligent and able to acquire information from the environment and reason about it.

Such an emerging environment, when carefully structured and efficiently used, is considered to offer significant opportunities to positively impact human life, both at an individual and at a societal level, and in particular to provide useful means to support people in their daily life activities and thus improve well-being [52]. This can be achieved precisely by exploiting not only the single functionalities of each device or physical smart object, but through their interconnection and integration in appropriately designed “support systems” (referred to as “applications” in this paper) which fulfill specific human needs, and may be equipped with the necessary level of intelligence to adapt at run-time the provided support based on contextual parameters. At the same time, this paper claims that as contact with other people may be the only effective support in many cases, it should be supported through such applications.

It is evident that applications in intelligent environments are potentially important for all people, and hopefully this will drive their uptake in the mainstream market. However, they are anticipated to become particularly useful for older people and for people with limitation of activities.^1^ This large and diverse user group, with a variety of different physical, sensory, and cognitive capabilities, can benefit from technological applications which can enable them to retain or improve their health, well-being, and independent living, but at the same time they may have considerable difficulties in using new technologies. This implies that applications and their interfaces must be accessible, simple to use and adaptable to individual users, automatically or by the users themselves [17].

However, accessibility and usability are not sufficient to ensure that applications and services are appropriately designed to satisfy human needs and overcome potential functional limitations in the execution of everyday activities fundamental for well-being.

This position paper puts forward the claim that, in order to achieve the above objective, it is necessary that: (i) the design of *Assistive Intelligent Environments* is centered around the well-being of people, roughly intended as the possibility of executing the (everyday) human activities necessary for living (independently), thus emphasizing usefulness in addition to usability; (ii) the technological environment is orchestrated around such activities and contains knowledge about how they are performed and how people need to be supported to perform them; (iii) the environment makes use of monitoring and reasoning capabilities in order to adapt, fine-tune and evolve over time the type and level of support provided, and this process takes place considering ethical values; (iv) that the applications must also support the possibility of contact with other people, who in many cases may be the only effective help.

The above requirements are exemplified and illustrated through the experience acquired in the development of four applications, addressing vital aspects of human life, namely *nutrition*, *stress management*, *sleep management*, and *counteracting loneliness*.

Following a brief overview of the evolution from accessibility and usability to Design for All, this paper discusses how the latter can be revisited under the perspective of technology’s usefulness and contribution to human well-being. Subsequently, it introduces a practical notion of well-being based on the ICF classification of human functions and activities and discusses how such notion can constitute the starting point and the focus of design approaches targeted to assist people in their everyday life mainly (but not exclusively) in the home environment. As a subsequent step, the need for integrating Artificial Intelligence capabilities in assistive applications is discussed, based on the complexity of the human problems to be addressed and the diversity of the types of support needed. The four above-mentioned applications are then briefly described, highlighting the aspects of the outlined approach that they address and the obtained results. Finally, based on the acquired experience, the need to take into account ethical values in the development of assistive intelligent environments is briefly discussed.

## People and ICT–From accessibility to intelligent living environments

### From *accessibility* to *design for All*

Initially, ICT development was targeted to support remote communication and access to information, through the availability of suitable equipment (from the telephone to computers and smartphones) and software. From the user perspective, one of the concerns which emerged with the wide adoption of ICT was to ensure that the use of the equipment was possible for all potential users, i.e. to ensure accessibility. For example, the telephone was not accessible for people who were not able to hear and the computer screen was not accessible for people who were not able to see.

The first research efforts pursued accessibility via a posteriori adaptation, that is, by employing assistive technologies to provide access to applications that were originally designed and developed for non-disabled users [49]. Specific adaptations were developed to solve these specific problems, a typical example being the screen reader for blind people [16], [2]). The reactive nature of these approaches has been criticized for its failure to catch up with the fast pace with which technology evolves, for its cost-ineffectiveness, as well as for the fact that it cannot always ensure equal access without functionality loss [49]. This criticism stimulated the conceptualization of theories, methodologies and tools of a proactive and more generic nature that could more accurately adapt to the increased interactivity of new technologies.

At the same time, the paradigm of Human-centered design emerged in HCI [40], [58], ISO 1999; ISO 2010; ISO 2019). Human-centered design claims that the quality of use of a system, including usability, depends on the characteristics of the users, tasks, and the organizational and physical environment in which the system is used. It also stresses the importance of understanding and identifying the details of this context in order to guide early design decisions and provides a basis for evaluation. While human-centered design focuses on maintaining a multidisciplinary and user-involving perspective into systems development, it does not specify how designers can cope with radically different user groups such as people with functional limitations or old people.

A general approach to designing for accessibility was then advocated by researchers, institutions and users: the Design for All approach. Design for All in HCI [48–50] is rooted in the fusion of human-centered design, placing the users as human beings at the center of the interaction design process, accessibility and assistive technologies for disabled people, and Universal Design for physical products and the built environment. The term Universal Design was coined to describe the concept of designing products and the built environment to be both aesthetically pleasing and usable to the greatest extent possible by everyone, regardless of their age, ability, or status in life [32]. Although the scope of the concept has always been broader, its focus has tended to be on the built environment. In the context of HCI, Design for All has been defined as a general framework catering for conscious and systematic efforts to proactively apply principles, methods, and tools to develop IT products and services accessible and usable by all citizens, thus avoiding the need for a posteriori adaptation, or specialized design [48].

As a result of the above efforts, the importance of accessibility is nowadays globally recognized, not only for people with activity limitations, but for anyone, as people’s abilities are constantly changing, for example due to age or to situations of temporary functional limitations. The Design for All approach was very successful towards elaborating and adopting technical recommendations (for example the W3C-WAI guidelines for the accessibility of the World Wide Web [59, 60]. Such guidelines constitute de facto standards, as well as the basis for legislation and regulation related to accessibility in many countries [25].

However, industry at large, while paying progressively more attention to accessibility, has so far not embraced proactive approaches. A potential reason that has been identified early on is that, although the total number of persons with functional limitations is large, each individual limitation represents only a small portion of the population; therefore, it would be impractical and impossible to design everything so that it is accessible by everyone regardless of their limitations [56]. On the other hand, the range of human abilities and the range of situations or limitations that users may find themselves in is too large;therefore, products could only focus on being as flexible as commercially practical [57]. Additionally, as proactive approaches do not advocate a “one-size-fits-all” approach, but aim instead to promote accessibility for everyone through the adaptation of the design to each individual user, companies may perceive Design for All as an extra cost or an extra feature. Such perception, however, is not accurate [35]. On the contrary, it has been claimed that by adopting design for all approaches, companies could achieve a number of business-oriented objectives, including to increase market share, take market leadership, enter new product markets, achieve technology leadership, and improve customer satisfaction [14].

Overall, proactive approaches do not propose the elimination of assistive technologies. On the contrary, such technologies have always been a good solution to many problems of individuals with disabilities. Besides representing a societal need, assistive technologies constitute a notable niche market opportunity [57], and according to current market, predictions assistive technologies are even expected to experience some growth [44]. Nevertheless, as technology evolves, proactive approaches are becoming a more realistic and plausible solution in the near future, in particular in the context of the emergence of intelligent environments [17, 33], where ad hoc accessibility solutions may not be feasible.

Additionally, the progress of HCI and the development of multimodal interaction approaches, although not explicitly targeted to the design of accessible systems and services, has made available transductions between different modalities (e.g. from text to voice, from voice to text, from images and their text decryptions), as well natural physical interaction techniques, such as for example touch (e.g. [21] and gestures (e.g. [42]). As many of the adaptations required for people with activity limitations are now available as part of the operating systems or as applications, it can be claimed that solutions to accessibility problems exist in most cases, even if not through a direct application of the design for all approach.

### Smart devices

**Fig. 1:**
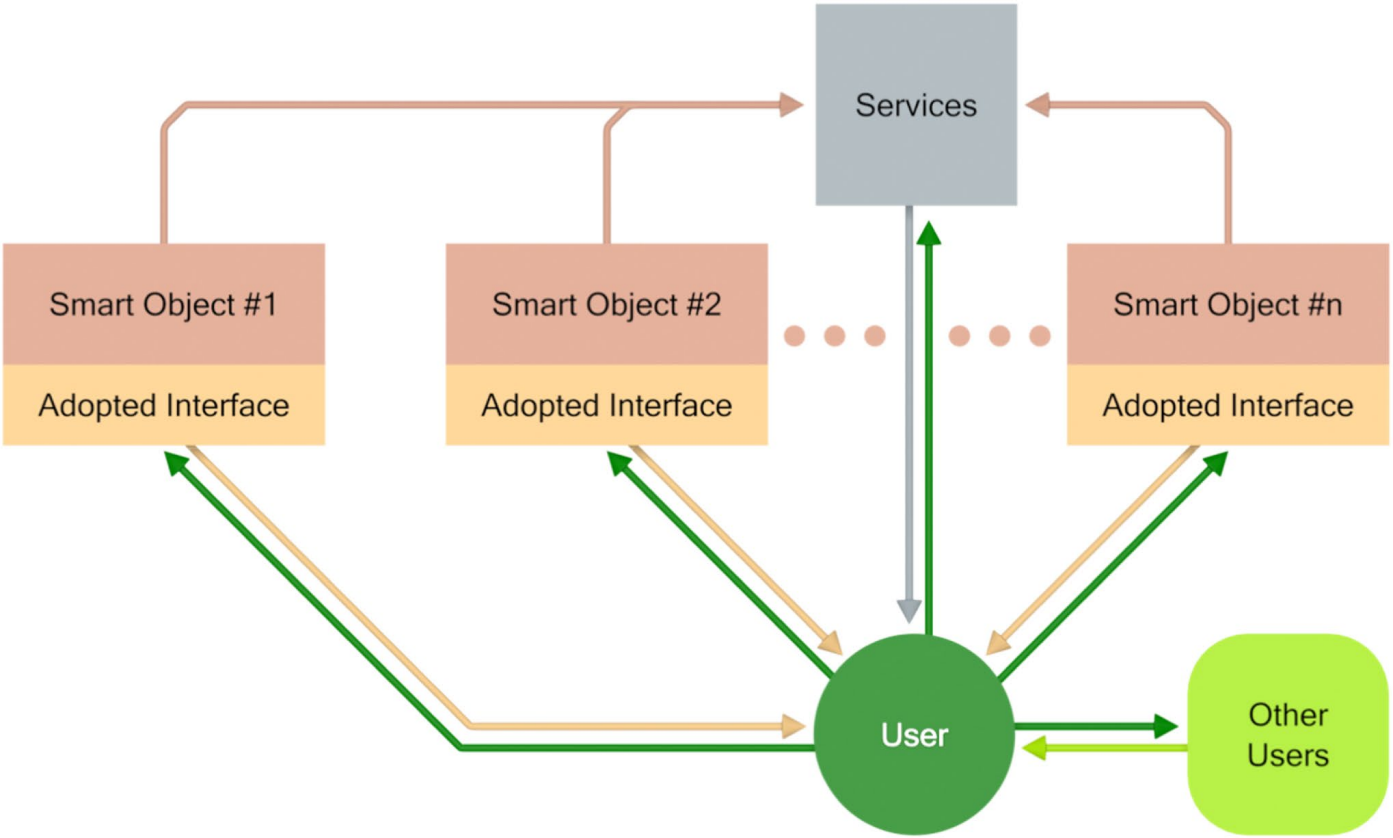
A smart environment (functionalities and services)

More recently, ICT development is also making available smart devices and objects that can be deployed in the environment, able to offer various types of independent functionalities [29]. An environment which includes such technologies can be represented as shown in Fig. 1, where the adapted interfaces are considered to take care of accessibility for all users. Usually, such adapted interfaces are not part of devices available on the market, though it is often easy to adapt the offered interface for the individual user, due to the facilities provided in the operating systems.

The model of Fig. 1 has found many useful applications for people in their living environments, such as, for example,^2^ communication and social interaction support (e.g. [38], monitoring of physical activity and health [36], and fall prevention (e.g. [24].

It is clear that most of the emphasis so far has been on problems related to safety, security and health care, which constitute fundamental prerequisites to independent life. These applications rely on sensor technologies, remote control and eHealth services. More recently, the*Ambient Assistive Living* (AAL) Programme has been launched in Europe by the EC,^3^ promoting the idea that ICT technology can be beneficially used in the environment to support people. AAL refers to intelligent systems of assistance for a better, healthier, and safer life in the preferred living environment and covers systems, products, and services that interlink and improve new technologies and the social environment, with a focus on older people.

### From usability of technology to usefulness of technological support systems

Figure 1 shown above reflects a type of system which can be designed following a classical human-centered approach. Some technology is available, such as for example sensors (e.g. for parameters connected to health care or security in the living environment) and actuators (for example for the use of home equipment), and care must be taken to make them accessible and usable, for example by adapting their interfaces for non-standard users [1]. In this paper, we maintain that there is the possibility of an approach moving from the needs of users, in particular in relation to everyday activities and well-being, and not from available technology. The underlying rationale is that people need support for carrying out activities in the emerging technological environment. Can emerging technology itself offer support to their needs? Such an approach is different from the well-known user-centred design paradigm [58], which aims to ensure that new technology is usable as an outcome of considering users in the design cycle. Indeed, it is more similar to approaches such as Value Sensitive Design (Friedman et al. 2013) and Need-based design [41], which aim to base the design of technology on the systematic analysis of individual and societal issues to be addressed.

In this case, we move on from the concept of Design for All and advocate an extension of its definition by not only addressing the accessibility and usability of technologies, but also placing emphasis on its usefulness, with reference to the entire population. This enhanced definition shifts the focus from the provision of accessibility and usability to anticipating the activities that people need to perform for living independently and comfortably, aiming to make such activities possible in ways which are suitable for individuals in dynamically changing physical and socio-psychological situations.

**Fig. 2:**
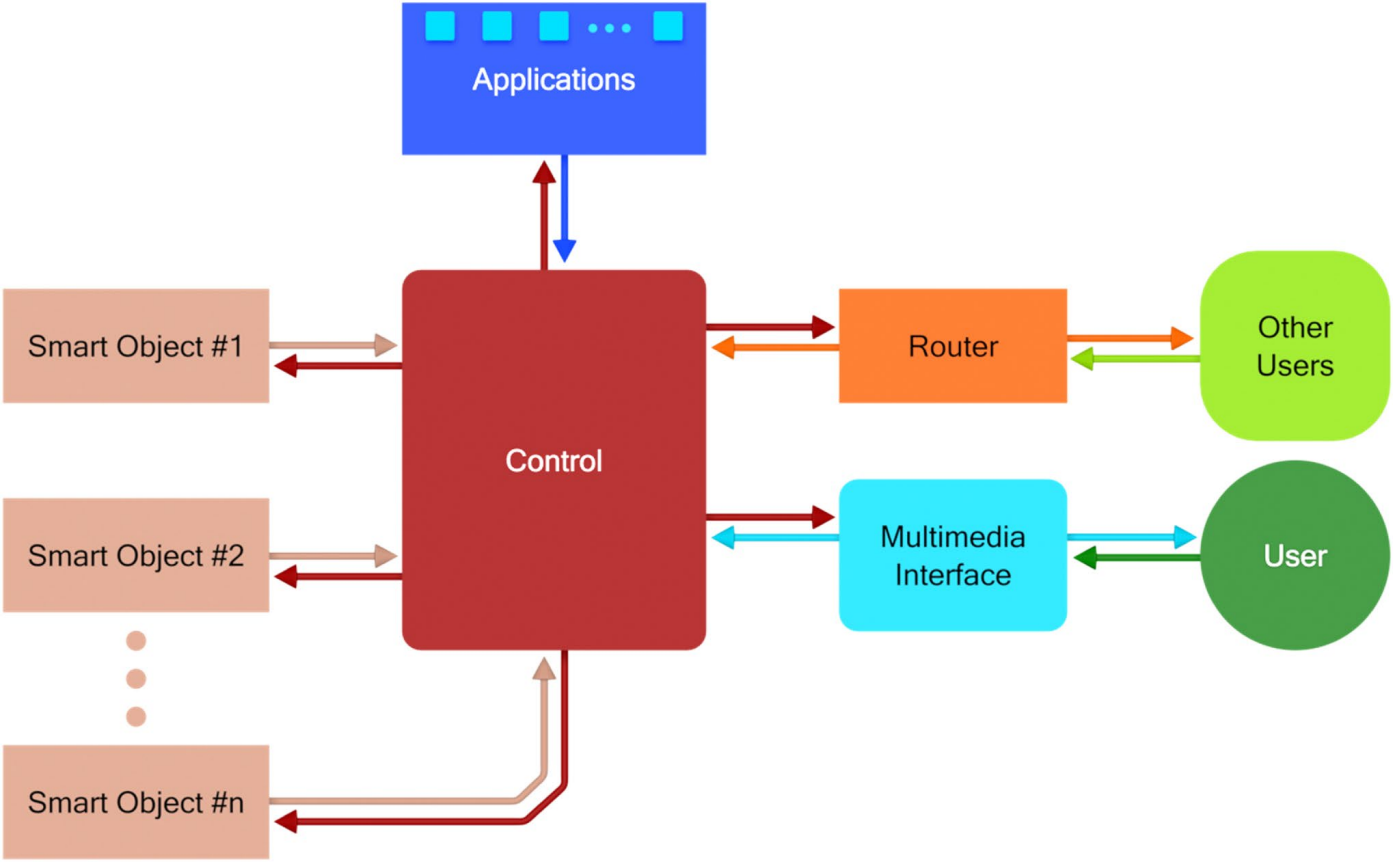
An integrated environment (application)

It is also argued that this shift has two main consequences. First, a richer architectural approach must be considered. The environment needs to be structured as shown in Fig. 2, where a control component is introduced. Devices and smart objects are not used in an isolated way, but are integrated to obtain a system able to support people in their activities, moving towards the direction of intelligent environments. An important characteristic of the scheme in Fig. 2 is that the users and not the technology are in the focus. The control is not seen from the perspective of the communication and interoperability of the technologies, but has the specific objective of supporting useful functionalities in the environment. In terms of design, the support to be offered is the main objective, to be pursued in two steps: (i) analysing the availability of suitable technology and the possibility of organizing it in a support system, (ii) designing the user experience and developing suitable user interfaces, taking also into account that people interact not only with the single devices, but with the (intelligent) environment [33].

The second consideration is that the environments to be designed should not be targeted to specific parts of the population, but to everybody, following the Design for All paradigm. This is very important also from the perspective of the market uptake of intelligent environments. It is more likely that in the near future couples of young people living together will have the financial resources for buying and the interest for “playing” with an intelligent house than an old person living alone. As in the case of transduction technologies, which have been mainly developed because of the interest of users of tablets or smart phones, this circumstance could contribute to elicit the interest of large industries, able to create a large market, thus reducing prices and increasing the possibility of use by all. If this is not the case, it is very likely that the proposed innovation will not have the foreseen success.

With respect to previous generations of technology, in the envisaged environment usability does not only imply ease of use, ease of learning and effectiveness in use of a system, but also the necessary adaptations of the service itself, because different people may need or prefer to carry out the same activity in different ways, or need to receive different types of information, as, for example, in the case of older people with mild cognitive limitations. The fact that people are not only supported by one or more devices addressing specific functionalities (is the blood pressure ok, did the person fall?), but also the different devices can cooperate, implies that complex support functions can be provided.

In this respect, the assistive technological environment goes in the direction of the requirements put forward by the ISTAG Group in 2001 and still actual today [15]:

- Facilitate human contacts;
- Be oriented towards community and cultural enhancement;
- Help to build knowledge and skills for work, better quality of work, citizenship, and consumer choice;
- Inspire trust and confidence;
- Be consistent with long-term sustainability–personal, societal and environmental—and with life-long learning;
- Be controllable by ordinary people.

The above characteristics are consistent with what is commonly advocated today in the study of intelligent environments: when dealing with people it is not necessary to address only their health care or other specific situations, but their overall well-being.

### Well-being and technology

It is advocated by many contemporary design approaches that intelligent technologies ought to be ethically inspired by human values and respond to individual and social needs [52]. In this position paper, we move from the starting point that, in a world where technology is omnipresent, the design of interactive systems should strive towards contributing to, and enhancing, human well-being, in demonstrable and measurable ways.

Subjective well-being is a growing area of research in social sciences, and in particular psychology. However, the concept of well-being is hard to define, and many of the existing approaches focus on identifying its components rather than defining it [13]. Two general perspectives have emerged over the years: the hedonic approach, which focuses on positive emotions such as happiness, positive affect, low negative affect, and satisfaction with life [11], and the eudaimonic^4^ approach, which focuses on meaning and self-realization, viewing well-being in terms of the degree to which a person is fully functioning [46]. In the latter tradition, various models have been proposed to capture the components of well-being. For example, the six-factors model [46] identifies autonomy, environmental mastery, personal growth, purpose in life, positive relations with others, and self-acceptance as fundamental aspects of positive functioning.

The hedonic and the eudemonic perspectives have given rise to a lively debate and have been seen in some cases as divergent and in others as complementary. Today, the common understanding is that well-being is a multi-dimensional construct involving both a hedonic and a eudemonic dimension. A related model is PERMA [47], which has been proposed in the context of Positive Psychology to support the concept of human flourishing, intended as ‘positive’ mental health [26]. PERMA includes five building blocks: positive emotions (corresponding to the hedonic dimensions), engagement (referring to an experience in which someone fully deploys their skills, strengths, and attention for a challenging task), relationships (insofar connections to others can give life purpose and meaning), meaning (as derived from belonging to and serving something bigger than the self), and accomplishment (pursue of achievement, competence, success, and mastery for its own sake, in a variety of domains, including the workplace, sports, etc.). Other recent approaches are based on evidence that life events significantly impact well-being, but people tend to maintain a balance and return to their usual well-being baseline after the occurrence of major events [13].

Many factors have been investigated with respect to their relevance for, and impact on, human well-being. Health is perhaps the most important. The relation between physical health and subjective well-being is bidirectional. People with chronic illness such as coronary heart disease, arthritis, and chronic lung disease show impaired hedonic and eudemonic well-being. On the other hand, well-being might have a protective role in health maintenance. In particular, eudemonic well-being is associated with increased survival [53]. Age is another interrelated factor. Subjective well-being is thought to remain relatively stable into old age despite health-related losses. However, studies have demonstrated that only some dimensions of subjective well-being remain stable, while others decline, and although age per se is not a cause of decline in subjective well-being, health constraints are [27]. Disability is another important factor. In particular, acquired disability has been found to be associated with moderate to large drops in happiness with little adaptation over time [30]. On the other hand, those born with a disability are likely to be happier as compared to those disabled later on in life. Happiness also decreases with the severity of disability, but appears to be independent of the type of physical disability [55]. Personality has also been found to be strongly associated with subjective well-being [31]. This might be due to the fact that temperament and other individual differences can influence people's feelings and evaluations of their lives, but also because people's emotions are an inherent part of personality. Specific personality traits are related to various types of well-being. For example, extroversion appears to be more strongly related to positive emotions, while neuroticism is more related to negative feelings. Although personality is an important correlate of subjective well-being, situations and life circumstances can in some cases have a considerable influence as well. Social relationships such as with family, friends, and partners are also considered as necessary for happiness [12]. With respect to culture, some types of well-being, as well as their causes, are consistent across cultures, whereas there are also unique patterns of well-being in societies that are not comparable across cultures [54].

With respect to the role of technology, it is generally assumed that it assists individuals in improving their well-being. However, the impact of new technologies and media on well-being and positive functioning is still somewhat controversial. In this respect, the quality of experience becomes the guiding principle in the design and development of new technologies, as well as a primary metric for the evaluation of their applications. ‘Positive technology’ [43] is a scientific and applied approach to the use of technology for improving well-being. Specifically, it suggests that it is possible to use technology to influence specific features of human experience that serve to promote adaptive behaviours and positive functioning, such as affective quality, engagement, and connectedness.

While we agree with the overall objectives of such approaches, in this paper we propose a different perspective, whereby a basic precondition for technologies to be able to improve human well-being, especially for the more vulnerable parts of the population such as the elderly and people with functional limitations, is that they positively support human functioning as defined in the WHO ICF classification.

### Well-being and human functions and activities

It is clear from the previous section that well-being is a very complex concept and a lively debate about its meaning and implications is in progress. In the context of this paper, we move from the consideration that well-being is strictly related to, and depends on, the possibility of conducting an independent, active, and fulfilling life. Therefore, we propose to base our approach on the WHO ICF classification of human functions and activities [61].

**Fig. 3:**
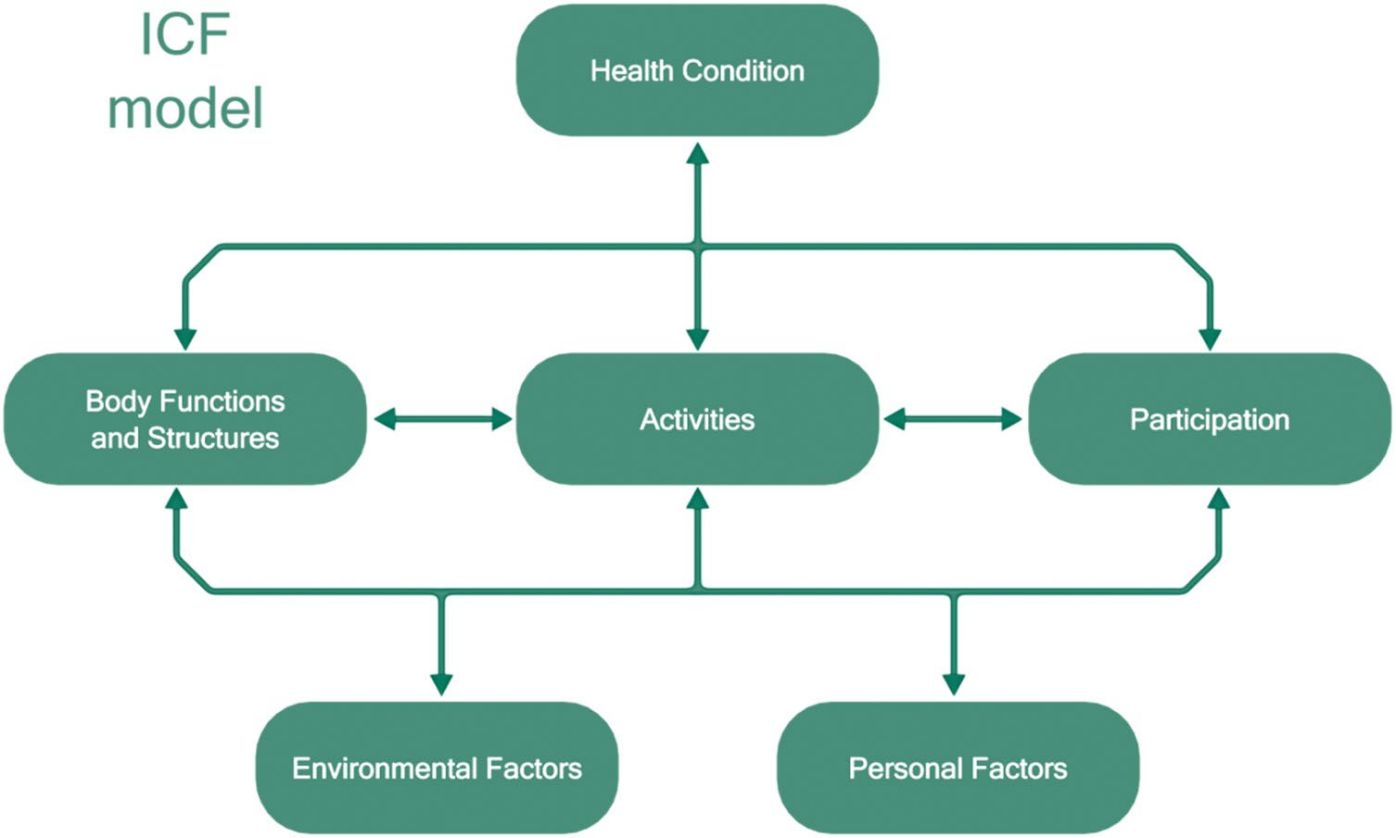
The ICF model

In ICF, the term functioning refers to all body functions, activities and participation, while disability is similarly an umbrella term for impairments, activity limitations, and participation restrictions. Body functions are defined as the physiological functions of body systems (including psychological functions). Activities are defined as the execution of a task or action by an individual. Activities may relate to the interplay of multiple functions and structures, i.e. functions and structures constitute preconditions of activities. ICF also lists environmental factors that interact with all these components. The functioning of an individual in a specific domain reflects an interaction between the health condition of the person and the contextual environmental and personal factors. Figure 3 depicts the ICF model.

A list of some activities as encoded in ICF is reproduced in Table 1, while the entire list of functions and activities is available in [60].

**Table 1:** Excerpts from ICF classification

D166 Reading
D450 Walking
D470 Moving using transportation
D550 Eating
D570 Looking after one’s health
D620 Shopping
D630 Preparing meals
D750–760 Informal social relationship
D910 Community life (organization)
D920 Recreation and leisure (sport, exhibition, hobbies…)

ICF also contains a list of the abilities necessary to carry out the necessary activities. Moreover, any activity is organized in a set of sub-activities, thus facilitating the identification of possible difficulties. Finally, the classification is conceived as open and can be refined up to the level of details that is considered suitable for each use.

We maintain in this paper that if intelligent environments are able to support people in everyday activities as modeled in ICF, this is at least a starting point towards improving their well-being. The classification of the necessary abilities is a support to reason about the possibility of performing the activities with the available abilities and to identify the necessary individual automatic adaptations of the environment and its interface(s), when some abilities are not available. It is intended that the listed activities must be carried out by all people and can be supported by mainstream technologies. Moreover, we recognize that support by technology may be useful, effective, and efficient, but in many situations support by humans is needed. This entails that applications should be based on or supported by social interactions, in order to allow for human intervention in the support loop.

**Fig. 4:**
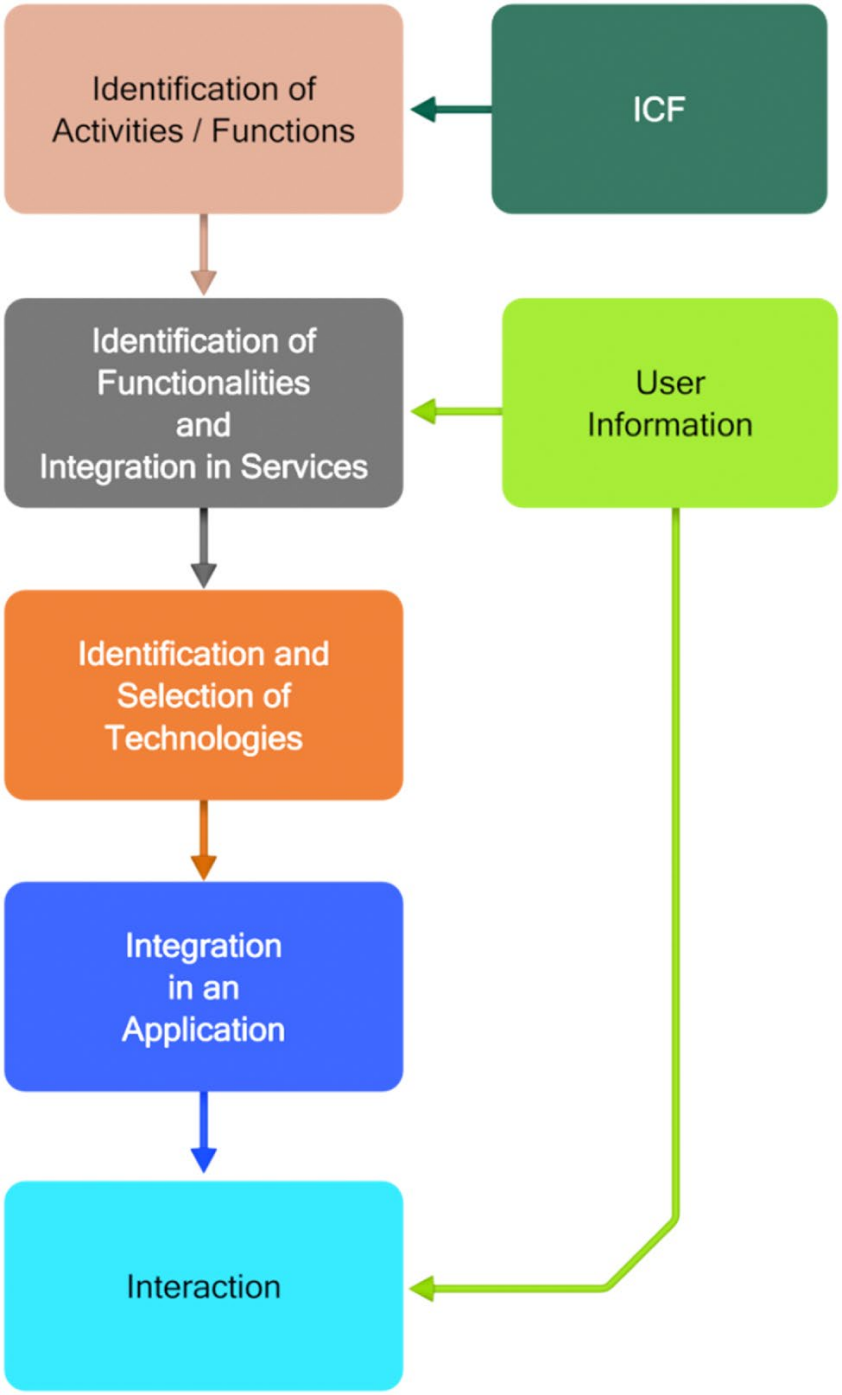
Structured Design procedure of an applications

A structured iterative process is necessary to elaborate user requirements and design applications based on the ICF classification. The one proposed in this paper is schematically shown in Fig. 4. The starting point is provided by the ICF classification. For example, people need to eat and for eating they have at least to acquire food, to know how to cook it and to be able to perform all the activities necessary for cooking it Table 1. The integration of these activities makes it possible to identify the necessary system functionalities (e.g. being able to switch on the oven) and services (e.g. if the person has manipulation abilities, a service must be made available to suggest the type of utensils to use) in order to overcome the functional limitations of the users. When this step is completed, the appropriate technological means (in terms of hardware and software) to provide the necessary functionality and services can be investigated and selected. This involves the selection of the devices and other physical objects to be deployed in the environment, as well as the identification of how their functionalities can contribute to the implementation of the necessary services. The entire environment should finally be organized in an application (or support service) able to satisfy the identified needs of target users.

The control component depicted in Fig. 2 is crucial in the proposed approach. Knowledge about the activities to be carried out, the necessary abilities, and the technological solutions to support all people, irrespective of their abilities, in carrying out the selected activity must be incorporated in the control unit.

The experience acquired during the development of the prototype applications described in this paper has demonstrated that the current level of technology development is adequate to support the development of applications following the approach described so far. However, there are problems which require the acquisition of knowledge from the environment, as well as reasoning about such knowledge, in order to be solved.

### Need for (artificial) intelligence

The experience acquired so far shows that useful supports to people can be offered using off-the-shelf hardware and mainstream software components, carefully integrated and orchestrated by the environment control system. Concurrently, other challenges emerge, that ask for the elaboration of a more powerful approach.

For example, if an application such as FOOD (described below in this paper) needs to recommend meal plans to persons with various types of health problems, one of the issues to be addressed is the complexity which emerges when the need for suggesting diets for people with pathologies connected to nutrition is considered. If the person is celiac, it is easy to suggest what food needs to be avoided. On the contrary, if the person is diabetic, the problem is complex because in addition to the knowledge of the health situation in general, it is necessary to consider real-time information as current blood sugar level today, what has been eaten yesterday and, if possible, what will be available in the near future. The situation may change in real time and differs from one person to another. Therefore, the system in principle should be able to adapt its recommendations to the real-time situation of any individual diabetic person. As a second example, we can consider an old person who begins to have mild cognitive problems that probably needs that all activities to be simplified according to the particular level of understanding that may change in real time (e.g. due to tiredness).

Awareness of real-time parameters can be achieved by monitoring through sensors. Typically, sensors monitor peoples’ daily activities at home, for example if they switch on the lights in a specific room, if they reduce their activity or fall or are depressed, etc. However, the environment is not normally able to connect patterns of use with possible problems. A caregiver, who knows the person’s background and monitors daily behaviour, is normally able to integrate this information. For example, she can succeed in understanding that the person has a reduced activity because in the morning she has carried out physically demanding activities, that she risks to fall because her pain in the back became worse after having walked for some time, or that she feels sad because she has been told that her daughter has some problems at work. It is probably possible and worth investigating whether the environment can, at least partially, mimic an informal caregiver not only acquiring and transmitting data, but also reasoning about them. Such a level of support is particularly relevant when psychological problems of people need to be considered.

Artificial Intelligence should be helpful in addressing problems such as the ones in the previous examples. There are many definitions of artificial intelligence [45]. Among them, the one considered in the present discussion refers to the idea that “the environment should be able to think “humanly”, i.e. it should be able to automate activities that we associate with human thinking, such as decision making, problem solving, learning …”. Moreover, the development of artificial intelligence solutions should not be carried out in a user-agnostic manner, instead it should be driven by the needs and requirements of users themselves, actively involving them in the loop [34].

**Fig. 5:**
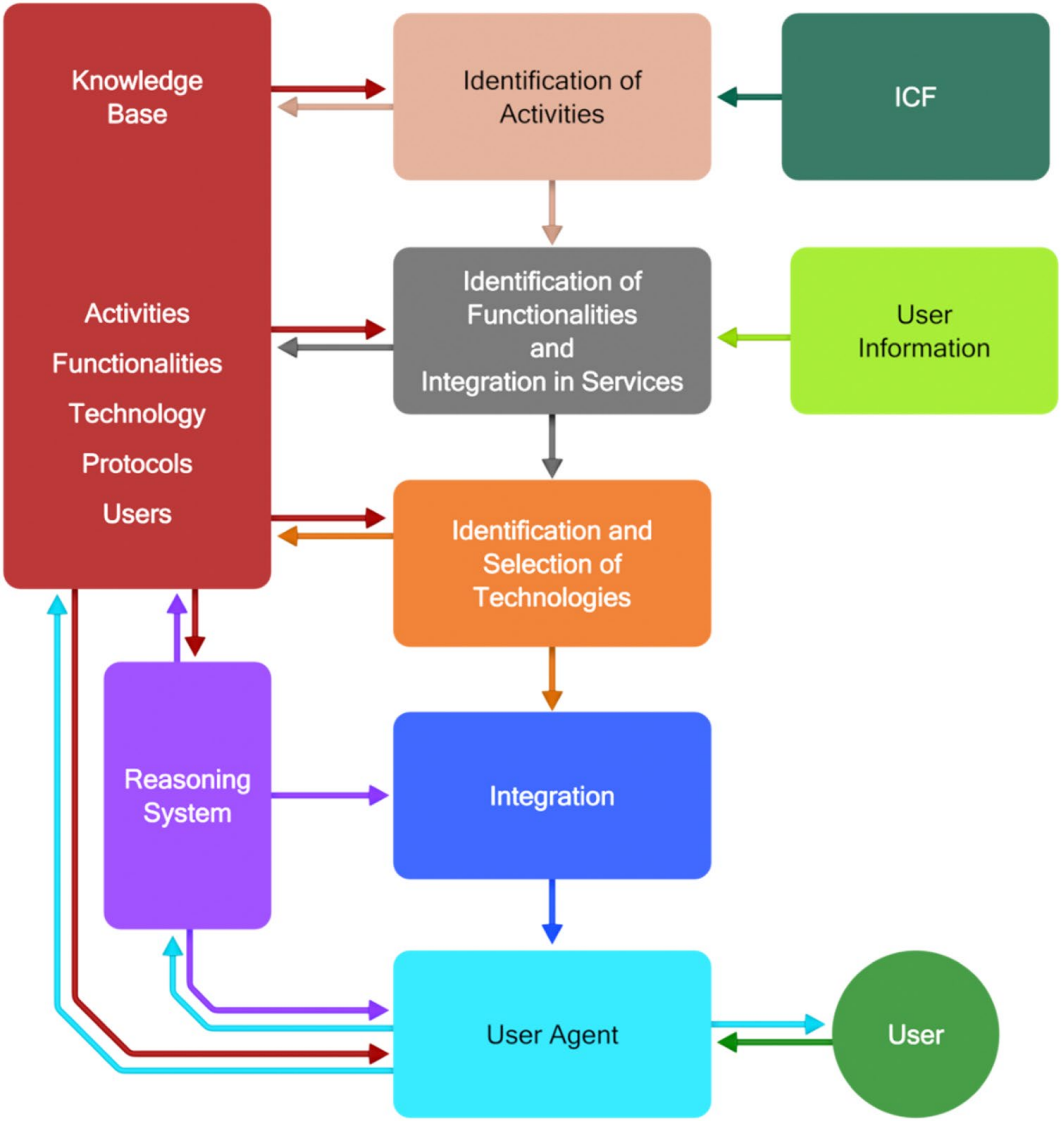
Implementation of an adaptable and adaptive application

Towards integrating Artificial Intelligence, the system abstractly depicted in Fig. 2 must evolve into the one depicted in Fig. 5 [4], where the control component consists of two main blocks: a knowledge base and a reasoning system. The following considerations are provided at a conceptual level, as they are independent of any specific implementation approach to be followed (beyond the scope of this position paper).

Irrespectively of how it is implemented, it is necessary that the *knowledge base* contains information about:

- The activities to be carried out in the environment;
- The functionalities, whose use either individually or in combination with other functionalities in complex applications is necessary to carry out the necessary activities;
- The technologies, whose basic functionality and embedded intelligence can contribute to the implementation of the functionalities;
- The interoperability issues, i.e. interfaces and communication protocols for the technologies to be used in an integrated form;
- The user profiles: abilities of individual users and their requirements and preferences regarding the functionalities to be used to support them in carrying out the necessary activities with the preferred interaction devices and modalities;
- The interaction issues: available interaction devices and modalities.

The produced knowledge must be structured appropriately according to the requirements of the reasoning components. Several technologies can be available for the implementation of the selected functionalities. The features of such technologies must be described in a formalized way together with the communication protocols. After a careful selection, all the collected knowledge needs to be integrated.

The implementation, maintenance, and run-time use of the system must be carried out under the control of a reasoning system (intelligence in the environment) capable of:

- Enriching the knowledge base, acquiring, and integrating information already received in a formal representation (rule-based AI) and/or extracting it (machine learning) from informal information (for example from natural language text);
- Using the information in the knowledge base to adapt, in an unobtrusive and anticipatory way, the functionalities made available in their interfaces;
- Learning from usage to refine the knowledge base, introducing the necessary updates;
- Evolving the functionalities according to the evolution of technology;
- Suggesting to the user alternative suitable means of interaction.

The adaptability of the intelligent environment to users’ requirements and preferences and its adaptivity to the changes in their behaviour, or in the context, was already advocated in the Design for All paradigm [6, 17, 51]. However, under the enhanced perspective proposed in this position paper, these concepts are expanded, as adaptability and adaptivity are not limited to interaction or merely based on deterministic rules (such as, for example, if the user is blind, then use voice and sound interaction). Instead, intelligent environments need advanced reasoning capabilities for identifying the goals of the users and helping them fulfill such goals using the available resources. The above mentioned characteristics comply with the requirements put forward in Ducatel et al. [15], and the resulting intelligent environment should be:

- Unobtrusive (i.e. many distributed devices are embedded in the environment and do not intrude into our consciousness, unless we need them);
- Personalized (i.e. it can recognize the user, and its behaviour can be tailored to the user’s needs);
- Adaptive (i.e. its behaviour can change in response to a person’s actions and environment);
- Anticipatory (i.e. it anticipates a person’s desires as much as possible without the need for mediation).

A fundamental issue in intelligent environments is the interaction with the environment itself and the services made available by it. In this context, there is an emphasis on “natural” user interfaces [20]. Although this paper does not aim to discuss in detail the meaning of this term, we can assume that it means interactions based on modalities and media typical of human–human interaction (e.g. using speech, the body language, gestures, facial expressions and so on). Under this perspective, there is a fundamental difference between professional environments (where people are supposed to learn how to use interactive systems) and the home environment, where the interaction that an individual considers “natural” in a given situation should be offered. For example, a person, probably not living alone, could consider natural to interact with the house in the same way as with other people, i.e. speaking and listening to answers, using body language and facial expressions. If images and videos need to be shown and the person does not want to go about the house using a tablet, they could be projected on any nearby flat surface. Obviously, if privacy is needed, then all information should be transferred to a device such as a tablet, accessible only to her. Therefore, as far as interaction is concerned, the intelligence in the environment should essentially be used to “understand” what the meaning of “natural” for each individual user is, on the basis of some initial information, as well as on continuous observation of how the individual user behaves. Moreover, the environment should also be able to mediate among the preferences of different users living together. Trends of developments in information technology and artificial intelligence (see, for example, the SIRI applications by Apple^5^ and the IBM Watson project^6^) show real possibilities of a successful evolution in this direction.

Finally, social networks can be fruitfully embedded in the very fabric of the intelligent environment. The information coming from social networks and any other application such as a forum, if conveniently processed, may contribute to the available knowledge. For example, when people at home are carrying out different activities, for example cooking, they could be very interested in speaking with friends for suggestions about recipes, in being advised by them when problems arise, in contacting the shop near home to get a missing ingredient and asking in the neighbourhood if someone can collect it.

## Developed applications

This section describes four applications which are partly (and to different degrees) inspired from the approach put forward in the previous sections, and show some of the characteristics discussed as prerequisites towards the provision of intelligent support to all. These applications have been developed in different contexts and with different aims, but they show a clear convergence towards the type of system envisaged in the previous section.

The first application, named *FOOD*, started from the request of the industrial partner of an AAL European project dealing with the smart kitchen [5]. They requested the development of an application that could help old people to prepare their food in the intelligent kitchen as a useful environment also for this user group. The main specifications given were the integration of available equipment with an application based with up to date industrial software. The tablet was the specified technology for interaction. The task was successfully performed, as shown by the evaluation described in the following section. However, it was also useful to understand some limitations. For example, it was sufficient for feeding, i.e. to help people to choose food and to help them to cook it, but not for nutrition, i.e. when a person needs the suggestion of a specific diet due to health problems and/or specific situations.

*CaLmi* (Sykianaki et al. 2019) and *HypnOs* (Tsolakou et al. 2020) are two applications which exemplify how intelligent environments can monitor the physiological and psychological status of their inhabitants and propose interventions targeted to improve well-being. They include reasoning components which are used to detect specific states from monitoring data (e.g. high levels of stress, poor quality of sleep), recognize problematic situations (e.g. stressful events, behaviours which hinder sleep quality) and perform decision making leading to personalized recommendations.

Finally, *Never Again Alone* is the first attempt of applying the architecture of Fig. 5 in the design of an intelligent support system targeted to alleviate the loneliness of elderly people through a social approach. The system is group-based, and is able to recognize the solitude of a component of the group and to recommend her activities (e.g. switch on the television) or to contact other members of the group, if available.

### Food

**Fig. 6:**
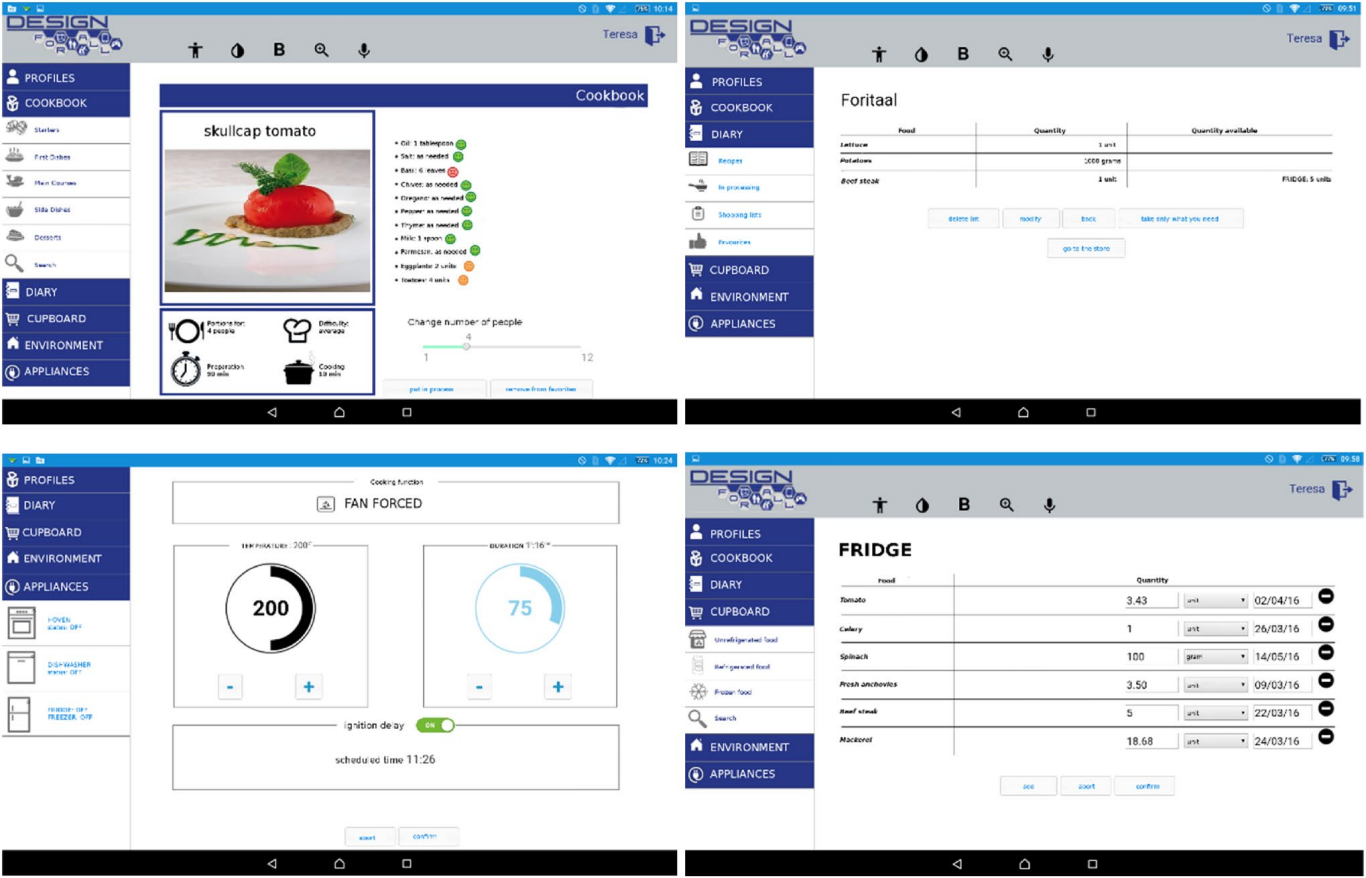
Snapshots of the FOOD user interface

The FOOD application [5] (Fig. 6) addresses a basic human activity, i.e. feeding. In Chapter 6 “Domestic Life” ICF identifies specific activities involved in the preparation of complex meals (d6301). The definition includes the following sub-activities: planning, organizing, cooking and serving meals with large number of ingredients that require complex methods of preparation and serving: this includes planning a meal with several dishes, transforming food ingredients by combined actions of peeling, slicing, mixing, kneading, stirring, and presenting and serving food in a manner appropriate to the occasion and culture.

The FOOD application is structured according to the block diagram of Fig. 2. The control block contains the software for the implementation including the rules for adaptation to the users, which have been produced by experts with an iteration of the procedure presented in Fig. 4. The personalization of the system’s behaviour to specific users is based on a set of information, which is provided (by the user or by a caregiver) when the user begins using the system.

In order to design an application able to support people in the management of the complexity of food acquisition and preparation, four main activities have been first identified. These are: (1) Selection of recipes (searched in an updatable data-base according to several criteria, as the name, or an ingredient or the type of course, e.g. dinner). The health status of the user and/or the availability of ingredients are considered, leading to the production of a shopping list modifiable by the user and to the management of the information about the content of the pantry; (2) Personal adaptations of recipes, to be memorized in a diary for personal notes; (3) Management of ingredients (available or to be acquired, occasionally or at regular intervals); (4) Use of necessary kitchen tools (e.g. knives) and home appliances for the preparation of the meal, mainly the oven, the hob and the fridge, including their maintenance. The next step is the preparation of the meal. In the system the recipes are structured in elementary steps and they can be presented in sequence. In each step the tools suitable for the user are suggested, if required. The cooking time is suggested and it may be transferred directly to the hob or to the oven. In the evaluation of the preparation time, the starting status of the ingredients is considered. For example, if an ingredient is frozen, the time necessary for defrosting is considered.

The FOOD application includes a user profile with the information necessary to help the users in selecting suitable food, preparing the dishes, and adapting the environment and the interaction. Information includes, for example, health situation, specific limitations of abilities, personal tastes, etc. In order to test some adaptations, three user profiles with minor limitations of abilities have been considered: Gianni (cognitive limitations), Teresa (manipulation limitations) and Sergio (low vision). For example, for Teresa, who has manipulation difficulties, a choice of the suitable tools is done, as knives that she is able to use. For Gianni (cognitive limitations), the recipes are simplified and their execution structured in an elementary form. For example, cooking an ingredient while preparing another one is avoided. If Gianni is not able to use personally the tablet, the screens may be projected on the wall. For Sergio’s interaction (low vision), a dedicated plugin for the inversion of the color and the magnification of portions of the screen has been developed.

A large variety of modalities has been considered for interacting with the tablet and with home appliances, such as vocal commands. Accessibility functions are made available in the App itself. Some facilities are implemented through the installation of a specific plugins.

The evaluation took the form of an (adapted) Pluralistic Usability Walkthrough [9]. To implement this approach, a group of stakeholders are brought together to review the design of a (testable) version of the prototype. In a typical PUW, representatives from at least three groups are involved in the evaluation: user experienced professionals (a psychologist expert in human–computer interaction), designers/developers, and users (not directly involved in carried out evaluation, but represented by two psychologists with a neuro-psychological training, who interpreted the users’ viewpoint, in order to allow an early detection of the potential usability issues that could affect real users). The evaluation process took into account the adaptations relevant to different user profiles, complex lifestyle, and human–environment interaction. The adopted procedure has demonstrated its validity and the usefulness and usability of the FOOD application [7]. It has also identified some aspects of the application to be improved for the future improvement of its usefulness.

### CaLmi

CaLmi (Sykianaki et al. 2019) is a system which exploits ambient technologies to assist those suffering from stress, an omnipresent problem of our times which has significant impact on people’s well-being [10]. Stress management is included in the ICF classification of activities under Chapter d2 “General Tasks and Demands”, d240 Handling stress and other psychological demands, which is defined as ‘Carrying out simple or complex and coordinated actions to manage and control the psychological demands required to carry out tasks demanding significant responsibilities and involving stress, distraction, or crises, such as driving a vehicle during heavy traffic or taking care of many children'. In particular, d2401 Handling stress is defined as ‘carrying out simple or complex and coordinated actions to cope with pressure, emergencies or stress associated with task performance’.

CaLmi is a pervasive system for intelligent homes that aims to reduce the stress of their inhabitants, thus contributing to the objective of improving their well-being. CaLmi’s architecture is similar to the one depicted in Fig. 2. However, instead of a unique control component, CaLmi includes two main modules, a stress detector and a recommendation system. The first is in charge of determining the levels of stress of users based on monitored physiological signals, along with information from the user profile and the current context. The second is in charge of recommending suitable personalized relaxation programs.

In case of stress detection, relaxation programs are selected based on the user’s preferences and the current context, and the user is recommended to start it. Available programs have been developed based on validated approaches to stress management and include for example exposure to nature, meditation and listening to relaxing music. Based on contextual data and observed behaviours, CaLmi can also predict which scheduled future event or situation might cause stress to the user and attempt to properly prepare her in advance. In order to accomplish that, CaLmi advices users when their upcoming schedule (according to the smart calendar) seems overwhelming to prepare ahead of time and thus to avoid last minute stress. Moreover, CaLmi displays general tips on reducing stress in daily life (e.g. do not drink too much coffee, eat healthy, sleep well, etc.).

CaLmi relaxation programs are adaptive, as they adjust to the user preferences and the current context of use (e.g. user’s current state, time constraints), while the user can optionally (de-)activate available features.

**Fig. 7:**
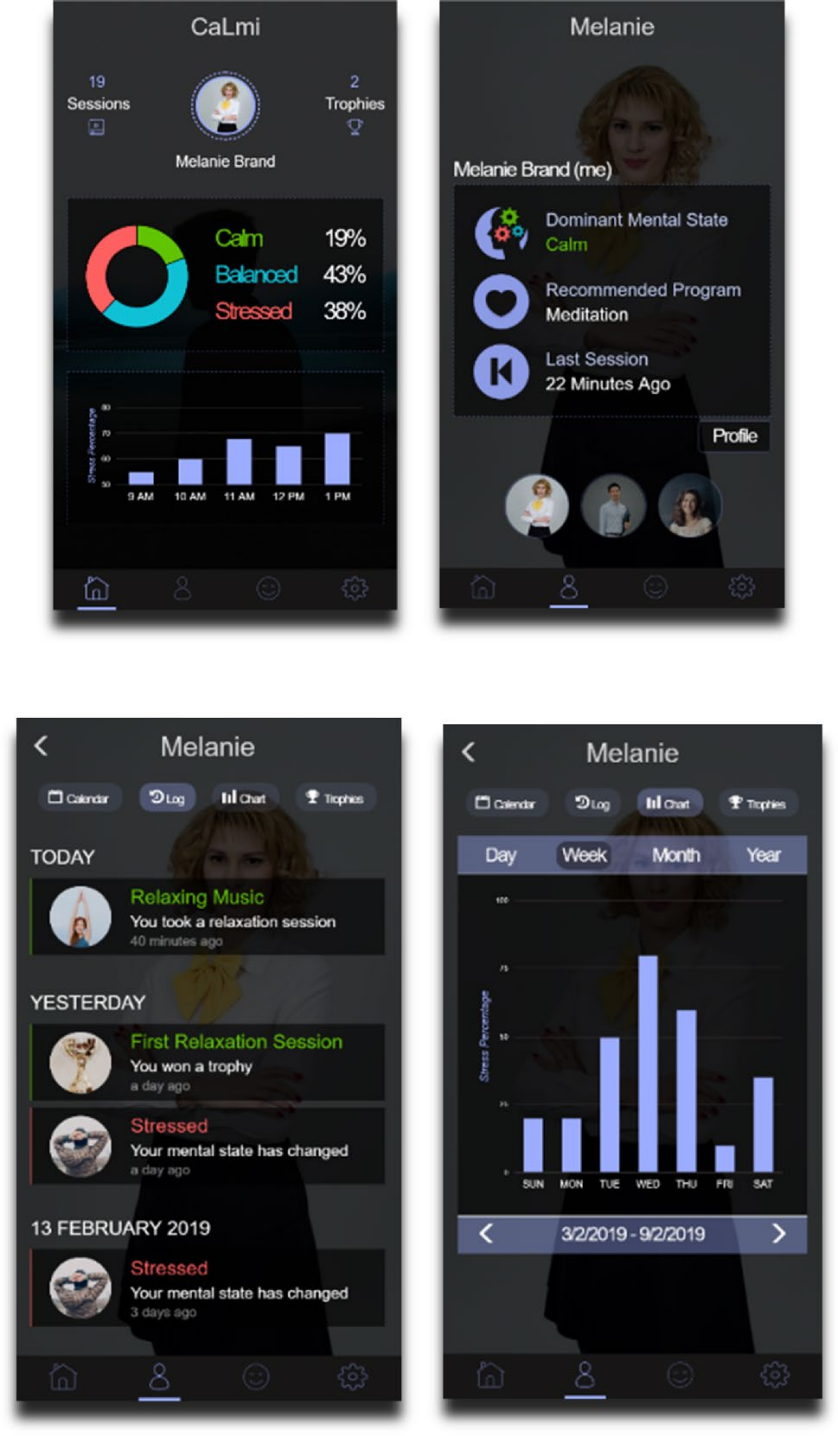
Snapshots of the CaLmi mobile application

A pervasive relaxation player application has been created to display the relaxation programs. By exploiting the home’s ambient facilities, the player can create a relaxing experience in the home environment. In particular, the player can project multimedia to a room’s display areas (e.g. wall, TV), play sound and music from the room’s speakers, adjust a room’s lighting conditions (e.g. light temperature, color, intensity), release a pleasant smell using a scent diffuser, etc. CaLmi also employs the pervasive notification system of the intelligent home in order to communicate urgent information to the user, by displaying messages to the nearest smart device. Additionally, CaLmi visualizes the user’s stress related data through a mobile application (Fig. 7). To enhance persuasiveness, a gamification mechanism is also used to encourage and motivate users to relax.

The system uses the typical technological equipment and installations of an intelligent home (e.g. hardware facilities, monitoring and decision-making mechanisms, distributed micro-services), and decides on the appropriate device in the environment to host for the selected program. Using the ambient facilities of the intelligent home, CaLmi is aware of the user’s location inside the house and tailors the program so as to be presented using the available facilities (e.g. TV, speakers, Wall Projector) to create a relaxing experience. For the detection of stress levels, CaLmi employs the Empatica E4 wristband^7^ that collects user biometrics.

Custom domain-specific micro-reasoners are used to identify stressful situations or behaviours. CaLmi also measures and compares a user’s stress levels before and after each program execution in order to observe their effectiveness and improve its recommendations for each individual user.

CaLmi has been evaluated to assess if it really reduces users’ stress levels as recorded by the Empatica E4 wristband. A total of 10 users participated in the evaluation. After a preliminary data collection phase, the participants were requested to wear the Empatica E4 wristband for three days, and whenever they were feeling stressed to try a relaxation program in one of the two offered modes, a multisensory one using the technological equipment and installations of the intelligent home in order to activate different, and a monosensory one, using only visual stimuli, in random order for each participant. The participants continued to wear the Empatica E4 wristband during the sessions and one hour after their completion in order to record their physiological signals and thus possible changes in stress levels. After each session the participants filled in a questionnaire about the relaxation program.

The results pointed out that a relaxation session is more effective and satisfying in the multisensory mode than in the monosensory mode, thus confirming that intelligent assistive environments can contribute to enhance well-being. More specifically, 80% of the participants thought that they were less stressed after the multisensory session in comparison with the monosensory, while all participants felt more calm, satisfied, sleepy and pleased after the multisensory one. In addition, the monitoring of physiological signals revealed that 60% of the participants were calmer after the multisensory session in comparison to the monosensory, and while their signals increased one hour after the end of the session, it was still lower than the starting signals. Moreover, the participants stated that they would use CaLmi in their everyday lives in order to receive multi-sensory, context-aware, personalized innervations for stress reduction.

### HypnOs

Sleep is essential for optimal cognitive performance, physiological processes, emotional regulation, and overall well-being. In today’s fast-paced society, however, many people suffer from sleep-related problems, which have negative consequences on sleep quality and therefore on well-being. In ICF sleep is defined as function b134 ‘General mental functions of periodic, reversible and selective physical and mental *disengagement from one's immediate environment accompanied by characteristic physiological changes’*. Sub-functions of sleep include b1340 Amount of sleep, b1341 Onset of sleep, b1342 Maintenance of sleep, b1343 Quality of sleep and b1344 Functions involving the sleep cycle.

The HypnOS framework for intelligent homes aims at improving the sleep quality of home residents by monitoring their sleep and providing personalized recommendations to overcome sleep-related issues (Tsolakou et al. 2020). Its architecture corresponds to the scheme of Fig. 2, since a central control module is responsible for both assessing sleep patterns and recommending lifestyle modifications to improve sleep quality. In addition, HypnOS is equipped with an AI framework used to calculate sleep scores, generate personalized sleep insights, schedule smart alarms, and recommend relaxation programs.

In order to assess sleep quality HypnOS utilizes sleep-related parameters and bio-signals obtained from wireless sleep trackers (i.e. wearable activity tracker/watch, under-the-mattress sleep tracker, and EEG headband), as well as contextual information made available by the infrastructure of the intelligent home. In addition, a daily sleep diary is provided in order to collect information about residents’ habits and their subjective measurements. In total, about 40 parameters are taken into account by HypnOs, including sleep-related parameters (e.g. sleep duration, snoring activity), bio-signals (e.g. heart rate, breathing rate), daily habits (e.g. caffeine and alcohol consumption), subjective measurements (e.g. sleepiness feeling, subjective sleep quality) and contextual information (e.g. daily activities, nutrition).

HypnOS not only unobtrusively detects sleep abnormalities, but also gains insights about the causes of the residents’ sleep-related issues in order to act accordingly. Based on the detected patterns, users receive personalized sleep recommendations to raise their awareness in order to change their sleep habits and activate any of the system's sleep/relaxation programs in case that they have difficulties falling asleep, exploiting the facilities offered by the CaLmi system described in the previous section.

**Fig. 8:**
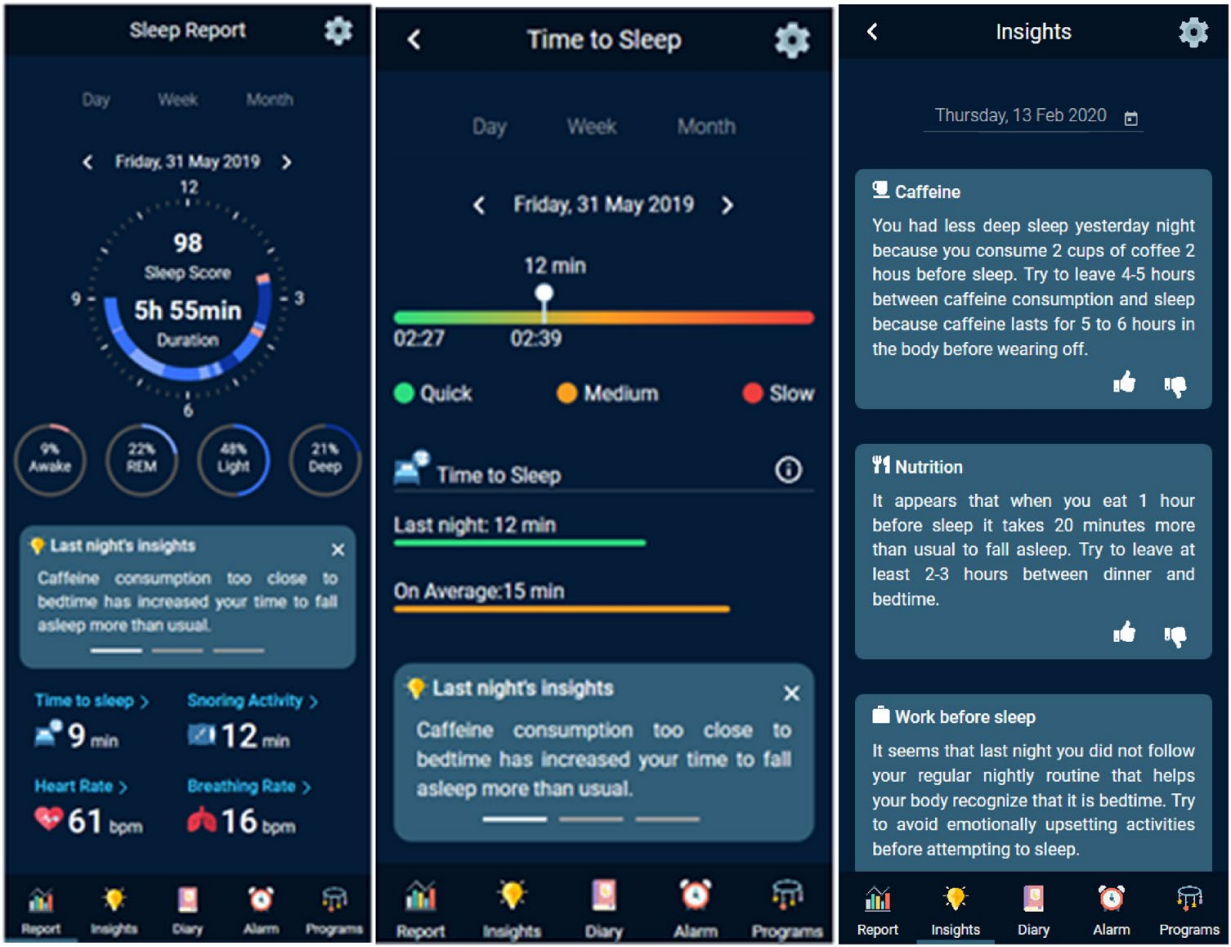
Snapshots of the HypnOS mobile application

HypnOs comes with a mobile application which visualizes detailed sleep reports including – amongst others – details regarding sleep patterns, movements during sleep, hours of sleep, time to fall asleep, snoring, etc (Fig. 8). Daily, weekly and monthly views are available. The application also offers personalized insights, a choice of relaxation programs, and the sleep diaries to be filled in.

HypnOS is deployed in the technologically enhanced bedroom of the intelligent home simulation space located at the AmI Facility within the FORTH-ICS campus, and utilizes the ambient facilities (e.g. lighting system, speakers, and scent diffuser) of the intelligent bedroom so as to enhance the environment (e.g. lights, sound, scent) and create experiences that can potentially facilitate the falling asleep and waking up processes. In particular, it offers a smart alarm which detects the optimal time to wake up users gently, and relaxations programs in cases where users have difficulty in falling asleep.

An observation-based user evaluation on the HypnOS mobile application has been conducted. The main objectives were to uncover potential usability issues and evaluate users’ overall satisfaction. In particular, the evaluation was targeted to assess the usability and perceived usefulness of HypnOs, the ease of getting sleep recommendations and viewing sleep-related information, and the willingness to complete the sleep diaries. Eight (8) users participated in the experiment. A custom observation grid was used to collect qualitative and quantitative information during the test. Two post-evaluation questionnaires were also used, aiming to reveal the usability of the mobile application, as well as the overall user satisfaction. The evaluation scenario included six tasks, which were given one by one to the participants.

The results were overall very positive, revealing that users can effectively use the application. Users were in their majority very satisfied with the mobile application, as a means to view their sleep statistics and to get personalized sleep recommendations. Users’ impression of the intelligent bedroom was also very positive. In particular, they were very enthusiastic about the sleep and wake up conditions that HypnOS is able to create (e.g. before sleep, bedroom’s lights simulated the sunset colors). Users also found the concept of HypnOS very useful and helpful. Also, it was verified that the majority of the users showed willingness to complete post- and pre-sleep diaries. Users would be also willing to wear / use all the sleep trackers (e.g. wearable activity tracker/watch, under-the-mattress sleep tracker) except from the EEG headband, which was found to be rather obtrusive. This brings about the need of using devices as sensors as unobtrusive as possible in ambient intelligence environments.

### Never again alone

The previous discussion about well-being highlighted the importance of psychological aspects towards living a healthy and fulfilling life. Among the emerging problems of our times, especially for elderly people, the relevance of loneliness is undeniable, and has acquired an even more dramatic dimension during the recent pandemic lockdown due the difficulties in personal contacts. In ICF, an entire chapter deals with interpersonal interactions and relationships (d7), as one of the main human activities. In particular, a section is specific for people living in the same building, d7501 “*Informal relationships with neighbours: Creating and maintaining informal relationships with people who live in nearby dwellings or living areas*”, since this represents a common life situation. Loneliness is considered to favor a large number of pathologies and functional limitations, especially with ageing. Several studies in different sectors, such as psychology, medicine, geriatrics state that loneliness may lead to heart attacks, depression and/or other pathologies [28].

The application is structured according to the scheme of Fig. 5 and assumes that, under a central control, it does not consider one person alone, but a group of users. It is based on a set of sensors and tools to acquire data about the user and the connected group of people to update in real-time the knowledge base, learning from the usage of the system. In return, the reasoning component suggests to the user one of the activities closely related to her interests and/or interests and present activities of other people in the group.

**Fig. 9:**
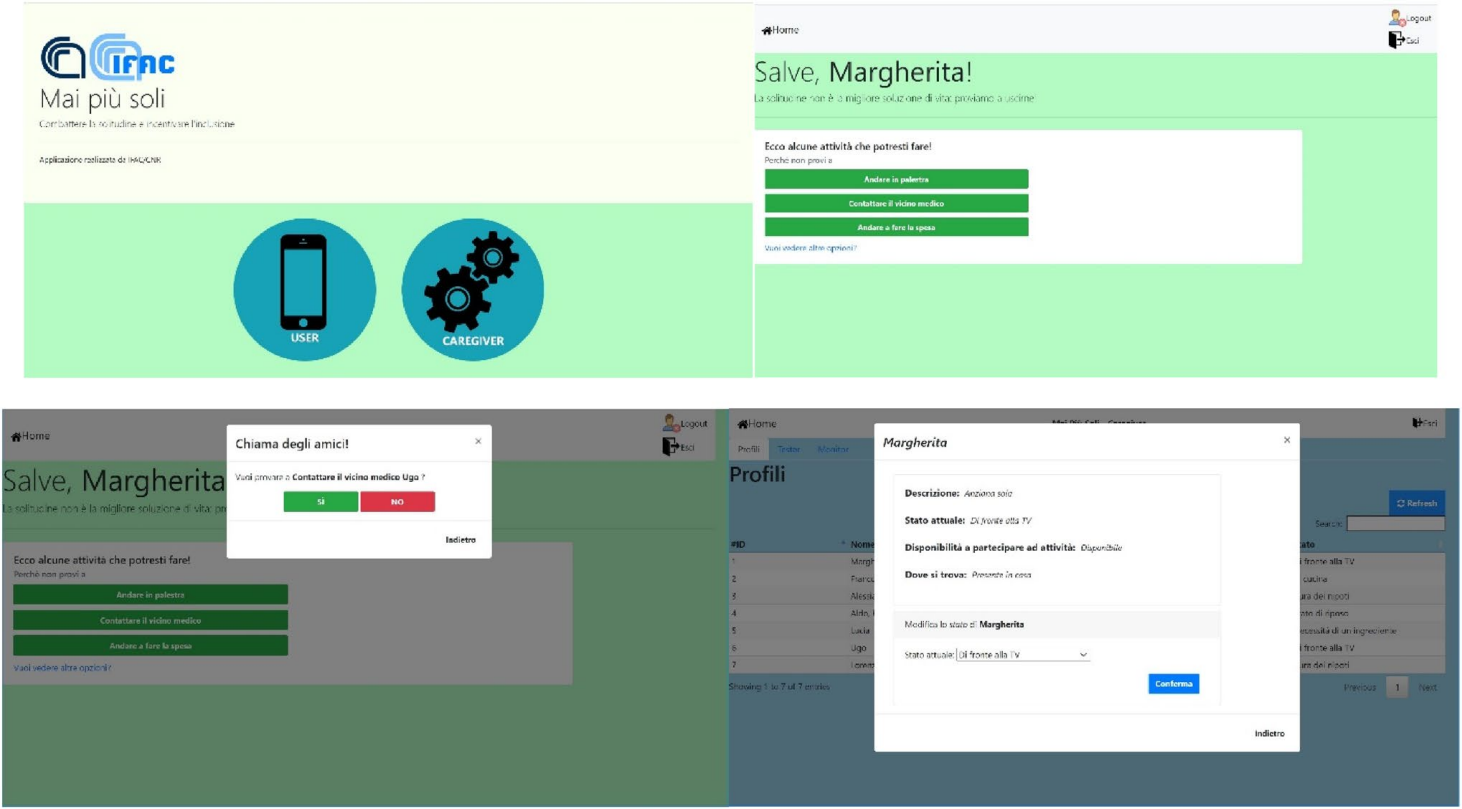
Snapshots of Never Again Alone

The design of a specific support system to counteract loneliness assumes particular relevance for the health and well-being of elderly people, who often live alone, although the phenomenon is not limited to them. The prototype support system Never Again Alone [8] (Fig. 9) provides support by offering recommendations of activities to reduce the corresponding negative feelings, in collaboration with other people. It is based on a group of people, living in the same building, who agree, according to privacy laws, to make available to the system knowledge of their main interests and of their present activities, obviously with real time control, and to support other components of the group, when necessary. This allows the identification of a specific and limited environmental context, in which relationships are mapped. The application is supposed to recognize the solitude of a component of the group and to recommend her activities (e.g. switch on the television) or to contact a member of the group, if available.

For the first implementation of the service, a particular context has been identified: a condominium. Seven different profiles have been identified and emulated in the system, including an elderly lady, an elderly couple, a couple with a baby, an elderly couple with a young grandchild, a young girl, a young doctor and an elderly man. When the system is started, the members of the group are represented by a static profile, in which their preferences assume a starting value. For example, whether or not they like cooking, football matches, or they have specific limitations of activities. Moreover, an initial set of suggestions is made available, in order to select, at the beginning within a limited but defined set, the best suggestion in each condition. The user profile and the first set of rules to select a suitable recommendation are based on experts’ evaluation and the experience of relatives, friends and care givers. In order to adapt the recommendations to specific users and situations, the system must be able to change its behaviour in a dynamic way, according to the variation of the behaviour of the user, her varying reactions to choices of activities in different situations and the current situation of the members of the condominium. Therefore, Artificial Intelligence techniques have been adopted in order to match different profiles, contexts and events. Expert systems and machine learning have been used to create the mechanism for feeding the data base of the system. The purpose is to provide each user with optimized results, depending on rules generated by the system itself and based on the reactions of people to previous suggestions. To this purpose, the machine learning algorithms consider the reactions of the user to accept the recommendations and the obtained results.

For the initial tests necessary to refine the implementation and to collect user reactions about their interaction preferences a tablet interface has been made available.

The idea and the initial specifications were presented to a group of students of Psychology of the Catholic University of Milan (Italy). The reaction was in general positive and interesting comments were offered by the students, indeed very useful to start the implementation of the system. Activities in a real context have also been planned with a direct involvement of users, in a public structure located close to Florence, (a cohousing structure with up to 80 people with a sufficient level of autonomy). A discussion with caregivers about the list of the most appropriate suggestions and methods to involve people is ongoing at the time of writing. The main emphasis is on guaranteeing the lack of invasiveness and the impact on favoring social life of the final system [8]. However, the current COVID 19 pandemic has slowed down the test phase that will be completed when it will be possible to contact the people in the cohousing structure without risk for them. An evaluation of the application in the Ambient Intelligence Research Facility of ICS-FORTH in Heraklion (Crete, Greece) is also planned.

### Ethics

The high level of autonomy in decision making that the systems described above can obtain moves the discussion of the design approach and set of requirements of such systems also towards ethical aspects. Ethical aspects of AI systems are raising a lively debate nowadays, addressing issues such as responsibility and legal consequences of actions and decisions made by an AI system, the integration of societal, legal and moral values into the of AI systems (Dignum, 2018), and transparency of AI decisions (Alaieri & Vellino, 2016). The above issues are relevant for all users, but may become critical when older people or people with functional limitations are involved.

Among various policy initiatives in this area, the European Commission has published in 2019 a reference document entitled AIHLEG ‘Ethics Guidelines for Trustworthy AI’ [18]. Three main components of trustworthy AI are identified: Lawful AI, Ethical AI and Robust AI. Only the last two aspects are considered in the document.

In particular, Ethical AI is based on four main elements: Respect for human autonomy, Prevention of harm, Fairness and Explicability, where explicability is a principle that enables the other principles through intelligibility and accountability. The four main principles are then translated in seven requirements: (1) human agency and oversight, (2) technical robustness and safety, (3) privacy and data governance, (4) transparency, (5) diversity, non-discrimination and fairness, (6) environmental and societal well-being and (7) accountability. All these requirements are then combined in a concrete, even if not non-exhaustive, assessment list of 131 practical questions. Such list represents a first tool in order to check the correspondence of a development activity with ethics criteria.

On the basis of the above, a process of analysis of the Never Again Alone application is ongoing, together with the comparison with other reference documents for Recommender Systems [37]. The aforementioned approach converges with a number of approaches proposed at an international level. For example, in [19], the authors show how it is possible to connect all the different principles presented by seven international publications to the four bioethical principles of beneficence, non-maleficence, autonomy and justice. The importance of the topics is further highlighted by initiatives such as the “Montréal Declaration for a Responsible Development of Artificial Intelligence” [39], the IEEE “A Vision for Prioritizing Human Well-being with Autonomous and Intelligent Systems” [23], or the IBM “Everyday Ethics for Artificial Intelligence” [22].

## Conclusions

This position paper has proposed an approach to the development of assistive intelligent environments based on an enhanced notion of Design for All, aiming to bind the degree to which intelligent technologies may satisfy human needs, thus aiming to contribute to well-being. Under such a perspective, the design of supportive environments is centered around the possibility of executing everyday activities as defined in the ICF classification of human functions activities. On the other hand, technological solutions need to satisfy specific technical requirements, i.e. they need to be orchestrated around the supported activities and contain the necessary knowledge in order to adapt the provided support to individual users. Finally, AI-based monitoring and reasoning approaches need to be followed in order to provide context-dependent run-time support and tackle complex activities of human life such as, for example, nutrition, stress management, sleep managements and addressing loneliness.

In order to exemplify the proposed process and its results, four developed applications addressing the above activities have been described. These applications satisfy (to different degrees) the above requirements towards the provision of intelligent support to all, and demonstrate that the approach is viable, as the developed applications are evaluated positively by the target users. More thorough evaluation studies are clearly required in order to assess longer-term effects, and are planned to take place as soon as the current worldwide pandemic will allow it.

The acquired experience in the development of these applications leads to a number of considerations. First, given the multifaceted and complex nature of human well-being, the provision of technological support to people is a highly multidisciplinary endeavor, which necessitates the involvement of medical doctors, therapists, psychologists, sociologists, and other professionals depending on the addressed problem (e.g. nutritionists, sleep experts, etc.). Second, important ethical problems arise when AI techniques are used. The relationship between ethical aspects and AI technology arouses great interest today, since the latest developments in artificial intelligence have produced results that affect society immensely. It is therefore particularly important that the ethical implications of technology-based support through intelligent applications are investigated and addressed.

Against the background of scientific contributions and technological developments for more than three decades in the fields of accessibility and usability, the authors of this position paper highlight important changes in the approach to addressing the challenges in this field: from accessibility to support of people, from usability to usefulness, from health care to well-being and from the pursue of technological developments to the pursue of effectiveness of the application of technologies made available, i.e. meeting the actual needs and expectations of people. The requirement of increased attention to ethics, mainly with reference to applications of Artificial Intelligence, has also been highlighted.

Our ongoing and future work will continue on the tracks identified in this position paper, further elaborating on the enhanced notion of Design for All in the context of intelligent environments, proposing requirements, methods and tools in support of the people living in, and interacting with such environments. More user evaluation studies of developed applications in this context will be carried out and a joint R&D agenda to intensify collaboration in this area will be also elaborated in pursue of developing technologies that offer valuable support to human life and well-being.

## Footnotes

1 According to the WHO International Classification of Functioning, Disability and Health (ICF), the term ‘people with limitations of abilities’ replaces the terms 'people with disabilities’ or ‘disabled people’. In such perspective, people are characterized by their abilities and by the lack of some of them.

2 The examples used for clarifying some of the statements in this paper refer to life at home and particularly in the kitchen. However, most of the presented observations are applicable in any living environment.

3 The AAL EC Programme (http://www.aal-europe.eu/about/) aims to foster the emergence of innovative ICT-based products, services and systems for ageing well at home, in the community, and at work; create a critical mass of research, development, and innovation at EU level in technologies and services for ageing well, including the establishment of a favourable environment for participation of SMEs; and, help create the market conditions for the industrial exploitation of healthy ageing products by providing a European framework that supports the development of standardized solutions and facilitates their adaptation to local, regional, and national levels to account for varying social preferences and regulatory requirements.

4 Eudaimonia is a concept that can be traced to classic Hellenic philosophy, and also constitutes a topic of contemporary philosophy. It stands for realizing one’s potential and is associated with several variables including self-determination, a balance of challenges and skills, and the investment of considerable effort.

5 https://www.apple.com/siri/.

6 https://www.ibm.com/watson.

7 https://www.empatica.com/research/e4/.
